# What Pre-clinical Rat Models Can Tell Us About Anxiety Across the Menstrual Cycle in Healthy and Clinically Anxious Humans

**DOI:** 10.1007/s11920-022-01376-7

**Published:** 2022-10-18

**Authors:** Jodie E. Pestana, Nusaibah Islam, Natasha L. Van der Eyk, Bronwyn M. Graham

**Affiliations:** School of Psychology, The University of New South Wales Australia, Sydney, NSW Australia

**Keywords:** Menstrual cycle, Oestrous cycle, Anxiety, Unlearned fear, Sex hormones

## Abstract

**Purpose of Review:**

Anxiety symptoms increase during the peri-menstrual phase of the menstrual cycle in people with anxiety disorders. Whether this reflects a heightened variant of normal menstrual-related changes in psychological states experienced by healthy (i.e. non-anxious) people is unknown. Moreover, menstrual-related change in anxiety symptoms is a poorly understood phenomenon, highlighting a need for pre-clinical models to aid mechanistic discovery. Here, we review recent evidence for menstrual effects on anxiety-like features in healthy humans as a counterpart to recent reviews that have focused on clinically anxious populations. We appraise the utility of rodent models to identify mechanisms of menstrual effects on anxiety and offer suggestions to harmonise methodological practices across species to advance knowledge in this field.

**Recent Findings:**

Consistent with reports in clinical populations, some evidence indicates anxiety symptoms increase during the peri-menstrual period in healthy people, although null results have been reported, and these effects are heterogeneous across studies and individuals. Studies in rats show robust increases in anxiety during analogous phases of the oestrous cycle.

**Summary:**

Studies in female rats are useful to identify the evolutionarily conserved biological mechanisms of menstrual-related changes in anxiety. Future experimental approaches in rats should model the heterogeneity observed in human studies to increase alignment across species and advance understanding of the individual factors that increase the propensity to experience menstrual-related changes in anxiety.

## Introduction

**Fig. 1:**
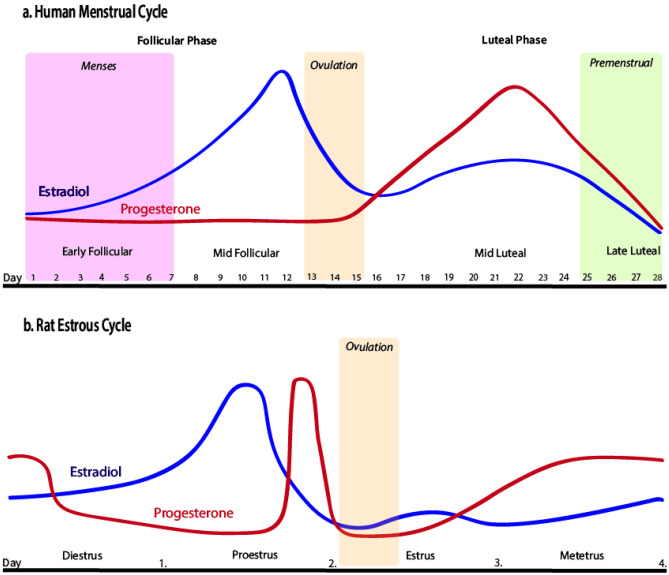
Sexually mature female rats and humans exhibit changes in secretion of the major ovarian hormones, oestradiol and progesterone, across their reproductive cycle, which are regulated by signalling from the hypothalamus and pituitary gland. Changes in these hormones over the average human menstrual cycle, which lasts approx. 28 days (notwithstanding substantial within- and between-person variability), are depicted in **Panel A**. At the start of the cycle (the follicular phase), oestradiol and progesterone are low, followed by a gradual rise in oestradiol produced by the developing follicle, triggered by elevated gonadotrophin-releasing hormone from the hypothalamus. Oestradiol surges prior to ovulation (the release of the egg), marking the end of the follicular phase, and then sharply declines. The luteal phase follows ovulation and is marked by elevated progesterone levels, and a secondary (smaller) elevation in oestradiol, secreted by the corpus luteum (the transient gland formed in the ovary after the release of the egg). Once the corpus luteum atrophies, progesterone and oestradiol decline; termed the late-luteal or premenstrual phase. In humans, the endometrial lining of the uterus thickens during the follicular phase; the reduction in hormones at the end of the luteal phase causes the endometrium to shed, which marks menstruation, or the start of the next cycle. The human menstrual cycle is typically measured using one or a combination of methods including calendar counting, self-reported onset of menstrual bleeding, luteinizing hormone tests for determining ovulation, and serum or salivary assessment of hormone levels. For recent excellent reviews providing recommendations for best practices in this field, see [4–6]. **Panel B** depicts typical fluctuations in oestradiol and progesterone across the rat oestrous cycle, which lasts 4–5 days. Oestradiol and progesterone are relatively low at the start of the cycle (termed ‘diestrus’, spanning two days often sub-divided into ‘metestrus’ and ‘diestrus’). The proestrus phase follows diestrus and is marked by an oestradiol surge, which occurs ~ 18 h prior to ovulation, as well as a progesterone surge, which increases sharply in the afternoon of proestrus (10–12 h prior to ovulation). Both hormone levels decline as the rat moves into oestrus, which is when ovulation occurs and the rat is sexually receptive (also known as the ‘behavioural oestrus’ phase). In rats, sexual activity is required to produce a functional corpus luteum. If no such activity occurs, the rats will move into metestrus the day after oestrus, and there is only a small increase in progesterone secreted by the corpus luteum which is evident on the evening of metestrus, and reduces on the morning of diestrus as the corpus luteum atrophies [7, 8]. The rat oestrous cycle is typically measured via visual inspection of vaginal cells [9]; corresponding hormonal levels are then inferred based on both vaginal cytology and the time of day the cells were collected. Note: oestradiol serum concentrations exist in the pico-molar range, whereas progesterone serum concentrations exist in the nano-molar range. These figures are based on data from [10–12]

The female menstrual cycle (Fig. 1A) modulates the severity and frequency of anxiety symptoms in a subset of menstruating people with a range of diagnostic subtypes, including generalised anxiety disorder (GAD), social anxiety disorder, panic disorder, posttraumatic stress disorder (PTSD) and obsessive–compulsive disorder, as summarised in recent reviews [1, 2, 3•].

Notwithstanding notable methodological limitations in many of these studies, there is relative consensus that anxious symptoms in this subset of people worsen peri-menstrually, i.e. the weeks prior to and during menstruation [3•], a phenomenon referred to as ‘premenstrual exacerbation’ (PME). PME is observed across different anxiety subtypes and symptom dimensions, including physiological [13], cognitive [14] and affective [15–17], and is not a product of comorbid reproductive endocrine conditions like premenstrual dysphoric disorder (PMDD). In most studies, PME is evident in a subset and not the full complement of anxious symptoms measured. Moreover, a minority of studies [18] find no evidence for menstrual changes in any symptom. Evidence supports substantial individual variation in PME [3•], akin to that reported for PMDD and perimenopausal mood disorder [19]. For example, based on retrospective reports, estimates of PME prevalence amongst menstruating people with anxiety or related disorders are 41–79% in panic disorder [20, 21], 45% in social anxiety disorder [17] and 48.5% in obsessive–compulsive disorder [22]. Whether menstrual fluctuations in anxiety symptoms are specific to people with anxiety disorders or are a heightened variant of normal changes in psychological states experienced over the menstrual cycle in healthy (i.e. non-anxious) people is an outstanding question. Moreover, the mechanisms underlying peri-menstrual exacerbation of anxiety symptoms are poorly understood (in anxious and non-anxious populations alike) and likely involve complex interplays between biological and psychological processes. Thus, pre-clinical animal models of menstrual changes in anxiety are required to enable the use of techniques that can identify biological mechanisms with greater precision and greater ability to interrogate cause-and-effect at a greater pace and scale than can be achieved in human studies. Promisingly, comparable changes in anxiety-like behaviour over the rat reproductive cycle (the ‘oestrous’ cycle) are well documented [23]. In this article, we review the evidence for menstrual changes in anxiety symptoms in healthy humans (i.e. those not meeting diagnostic criteria for a mental health condition, including PMDD) to provide a counterpart to the recent reviews on menstrual-related symptom changes in humans with anxiety disorders [2, 3•]. We also review the evidence for oestrous-mediated changes in anxiety-like behaviour in rats and compare these findings against those obtained in humans. We focus on rats because of the inconsistent oestrous-associated hormonal and behavioural changes reported in past literature in mice, possibly driven by strain differences and greater oestrous variability in mice [24–29]. We highlight both the complexities and the potential gains in investigating menstrual fluctuations in anxiety via a cross-species translational framework, and we offer recommendations for future research to harmonise methodological practices across species to improve our understanding of this phenomena in healthy and clinical human populations.

## Menstrual Fluctuations in Anxiety in Healthy (Non-anxious) Humans

Most research on menstrual changes in psychological symptoms in healthy people have focused on mood-related symptoms [30] or used global scales that average scores across distinct psychological states (mood and anxiety [31, 32•, 33, 34]), resulting in a minority of research that has expressly measured and reported statistical comparisons for changes in anxiety across the menstrual cycle in healthy people. These studies differ substantially with respect to anxiety dimension assessed, e.g. trait or state anxiety using validated measures [13, 18, 35–37] or single items [38], cognitive features of anxiety (repetitive negative thinking [14], perceptions of control [33, 34], anxiety sensitivity [39]), discrete emotions (nervousness/irritability [40]), sub-classes of anxiety (attachment anxiety [38], phobic anxiety [16], panic symptoms [13, 41, 42]) and fatigue [40, 43]. Moreover, most studies have assessed symptoms during pre-selected menstrual phases (e.g. mid-luteal versus early follicular) rather than over a full cycle, and the precise phases examined differ between studies. These factors make it difficult to compare findings across studies. Moreover, consistent with commentaries on menstrual cycle research more broadly [4, 44], many studies are methodologically limited. For example, most studies are underpowered (with sample sizes fewer than 10 in several studies), and menstrual phases are poorly defined and unreliably measured (e.g. via day count in the absence of tests to confirm ovulation). Likewise, measures assessing anxiety are often crude (e.g. a single item), and no study has measured the full range of physiological, affective, cognitive and behavioural dimensions that characterise anxiety. Finally, very few studies have directly compared anxious versus non-anxious populations, which is needed to determine whether the presence, quality or magnitude of cyclic changes in anxiety symptoms differs as a function of mental health status.

Despite the overall limitations of the literature outlined above, some studies support the existence of menstrual-related fluctuations in anxiety symptoms in healthy people. An early study [45] reported evidence for elevated premenstrual anxiety relative to ovulation in a thematic analysis of free association speech samples. Consistent with these findings, a later study [35] reported increased premenstrual state anxiety and obsessive–compulsive symptoms relative to the follicular phase, and these effects were independent of the severity of premenstrual physical symptoms reported (e.g. bloating, headache). A recent study [46•] in high-functioning trauma-exposed individuals, the majority of whom did not meet PTSD criteria, assessed PTSD symptoms daily across the follicular phase, as oestradiol levels were increasing, confirmed via salivary assays. They found that overall PTSD symptoms, negative cognitions, and arousal, decreased over this time interval. Additionally, oestradiol levels assessed during a lab visit negatively correlated with PTSD symptoms, and people in a high oestradiol menstrual phase (periovulatory, late-follicular) showed less sympathetic nervous system activity and more cortisol activity in response to a trauma reminder relative to those in a low oestradiol menstrual phase (early-follicular, early- and late-luteal). However, null results have also been reported in recent studies. One study [40] reported no menstrual-related changes in nervousness, irritability, or fatigue, assessed daily over at least two menstrual cycles, and another [36] reported no changes in state anxiety during the early- and mid-follicular and mid-luteal phases. Another study [38] reported no changes in attachment anxiety or avoidance over the follicular, ovulatory and luteal phases, but did find that as progesterone increased relative to each person’s average progesterone levels, anxiety also increased. Other studies [33, 34] found no changes in perceived control from the follicular to premenstrual phases. Studies using between-groups comparisons have reported mixed results [37, 38, 47], but a major limitation of between-group designs is the inability to control for individual differences in anxiety levels that could mask within-person variation.

Of the studies that have directly compared menstrual effects in anxious versus non-anxious people, some have reported no evidence for menstrual changes in self-reported anxiety symptoms, or panic symptoms, in non-anxious people [13, 18, 42], despite these studies finding some evidence for cyclic changes in anxiety symptoms in people with panic disorder. Likewise, a more recent study [48] reported no menstrual changes in panic symptoms in people without panic disorder, despite finding a premenstrual increase in panic symptoms in people with panic disorder and a menses-related increase in physical symptoms (e.g. bloating, headache) in both groups. We recently reported that non-anxious people exhibited increased mental fatigue from the early follicular to the mid-luteal phase, comparable to the high levels of fatigue exhibited by people with GAD at both menstrual phases [43]. In a separate study [14], we found no evidence for menstrual fluctuations in repetitive negative thinking and negative effect amongst non-anxious people, despite finding a mid-luteal increase in these symptoms in people with GAD. However, within-person fluctuations in progesterone were related to these symptoms in non-anxious people, but not those with GAD. Specifically, these symptoms were elevated during times of reduced progesterone levels relative to each person’s average, consistent with progesterone’s role in the synthesis of anxiolytic neurosteroids. Finally, studies on PTSD have reported an increase in phobic anxiety in the early-follicular phase relative to the mid-luteal phase in trauma-exposed people with PTSD, but not those without PTSD [16], with no cyclic changes in either group on anxiety sensitivity [39].

Other studies have examined individual difference factors that moderate menstrual fluctuations of anxiety symptoms in healthy people. Nillni et al. [41] compared changes in panic symptoms in healthy people who were high or low in anxiety sensitivity (fear of anxiety and anxiety-related sensations) at different menstrual phases. Cognitive responses to a CO_2_ challenge (e.g. fear of losing control) were higher in the premenstrual phase relative to the follicular phase, only in people high in anxiety sensitivity. There was no impact of the menstrual cycle in either group on physical or self-reported panic symptoms. A recent study [49] reported that whereas people higher in health anxiety reported increases in perceived stress from the follicular phase to the week prior to menses, the opposite pattern was noted in those lower in health anxiety. Notably, health anxiety did not moderate premenstrual symptoms, illustrating menstrual changes in anxiety symptoms seem to exist independent of premenstrual symptom severity.

Combined, evidence to date for menstrual-related fluctuations in anxiety in healthy people appears more equivocal than that in populations with anxiety disorders. However, of the studies that do report significant changes, in general (with some exceptions), it is the peri-menstrual phase that is associated with anxiety symptom exacerbation, consistent with the pattern of PME reported in clinical populations.

### Methodological Considerations in Studies of Menstrual-related Anxiety Change in Healthy Humans

The work by Nillni and colleagues highlights the importance of the moderating role of individual difference factors, such as anxiety sensitivity and health anxiety. These factors may be widely dispersed in a healthy population, as opposed to a clinical population which may cluster more uniformly around one point of these dimensions (e.g. high anxiety sensitivity). The wider dispersion of these factors in healthy populations could lead to greater variation in the magnitude, presence, or direction of menstrual cycle fluctuation and may partly account for null results or inconsistencies between studies in healthy people. Relatedly, menstrual fluctuation in anxiety symptoms is likely a heterogeneous phenomenon experienced by a subset of those who menstruate, and there may be distinct sub-patterns of cyclic changes in symptoms. This is exemplified in work by Kiesner [32•], who tracked changes in anxiety and depression in a community sample of 213 people (including those with and without psychiatric conditions and menstrual-related difficulties) across two menstrual cycles. A single score of anxiety and depression was calculated by collapsing responses to two items querying anxiety and four items querying depression in the last 24 h. Although the study is somewhat limited by the use of an unvalidated measure of anxiety, the outcomes highlight the importance of considering individual trajectories of symptom change over the cycle. Specifically, cluster analysis revealed four patterns of anxiety/depression change across the menstrual cycle, with one group reporting no cyclic change, another group reporting a midcycle symptom exacerbation (around ovulation), and the remaining two groups both reporting a ‘classic’ premenstrual symptom exacerbation, with one of the groups exhibiting a larger magnitude of change than the other. Combined, work by Nillni and Kiesner suggests that an advanced understanding of the phenomenology of menstrual fluctuation in anxiety symptoms in healthy people is likely to be made by using sufficiently sized samples to capture an array of individual variations and to use statistical techniques (e.g. latent group modelling) to identify subgroups exhibiting different patterns of symptom change. Conversely, groups could be selected for more focused investigations by pre-selecting individuals for heightened or reduced levels of moderators implicated in menstrual effects on anxiety (e.g. anxiety sensitivity, health anxiety).

Other methodological considerations include the importance of measuring for hormonal contraceptive use, like the oral contraceptive pill, which introduces high and relatively steady levels of synthetic oestradiol and progestins. The oral contraceptive pill chronically suppresses ovulation and inhibits ovarian production of oestradiol and progesterone. Several studies did not assess hormonal contraceptive use (e.g. [48]), which significantly compromises the internal validity of the study because changes in anxiety symptoms observed over a monthly timeframe in people taking hormonal contraceptives cannot be attributed to the impact of the menstrual cycle. Moreover, an underutilised but possibly useful approach is to compare changes in anxiety symptoms between cycling people and those taking the oral contraceptive pill. Such a design would enable hypotheses regarding the mechanisms of menstrual-related symptom change to be assessed. For example, if cyclic changes in hormones mediate symptom change, then the magnitude of symptom variance over time should be reduced in those taking the oral contraceptive pill (who have steady levels of hormones) relative to cycling people. A conceptually similar idea is to compare symptom change in cycling people during ovulatory versus anovulatory cycles, which differ in their hormonal profiles (e.g. anovulatory cycles have lower progesterone) and thus provide a comparison condition to enable the assessment of hypotheses regarding mechanisms of menstrual fluctuations in anxiety. For example, a within-person analysis in a longitudinal study [50] revealed a greater peri-menstrual increase in self-reported anxiety for ovulatory versus anovulatory cycles. Data from anovulatory cycles could easily be examined via within-person analyses over multiple cycles or between-person analyses over a single cycle.

## Oestrous Fluctuations in Anxiety-like Behaviour in Female Rats

Symptoms of anxiety disorders can be modelled in rats using unlearned fear paradigms. The most common unlearned fear tasks capitalise on the animal’s innate fear of ethologically relevant stimuli (e.g. elevated or open spaces, bright lights, predator odour) and therefore do not require training to evoke behavioural fear responses (e.g. avoidance, freezing), termed ‘anxiety-like behaviour’. These approach-avoidance conflict tasks include the elevated plus maze, open field test and light–dark box. Other unlearned fear tasks that do not depend on approach-avoidance conflict include those measuring active avoidance behaviour (e.g. shock probe burying test, marble burying test), startle response (e.g. acoustic startle, light-enhanced startle) or social behaviour (e.g. ultrasonic vocalisations, social interaction tests).

The impact of the oestrous cycle (Fig. 1B) on anxiety-like behaviour in female rats is conceptually consistent with findings in clinically anxious and healthy women*.* That is, female rats exhibit higher anxiety-like behaviour when tested during oestrous phases of low sex hormones (metestrus or diestrus) compared to oestrous phases of high sex hormones (proestrus). These effects are highly robust across various rat strains, testing paradigms, lighting conditions and experimental designs and have not diminished over time, with several recent studies reporting similar results [51–65, 66•, 67•, 68, 69•]. A minority report null effects [70–75]. The analogous findings cross-species suggest that changes in anxiety coincident with cycling hormones are likely linked to an evolutionarily conserved mechanism in female rats and women. Given that rats do not menstruate, it is unlikely that physical symptoms associated with menses in women (e.g. pain, physical discomfort) can completely account for peri-menstrual anxiety, notwithstanding the likely impact of social and cultural beliefs about menstruation [76]. For example, one possibility is that heightened anxiety during the premenstrual phase is a by-product of heightened threat perception following ovulation that facilitates a successful pregnancy [77], which may be more evident in hormonally sensitive individuals. Another possibility is that changes in anxiety coincident with cycling hormones across the reproductive cycle prepare rats and humans to respond appropriately to extended and elevated hormonal changes across the peripartum period to ensure offspring survival.

Potential biological mechanisms underlying hormonal-associated fluctuations in anxiety that have been examined in female rats and humans include oestradiol and the oestrogen receptors [78, 79], the progesterone metabolite allopregnanolone and its actions on the GABAergic system [80, 81] and the neurotransmitter serotonin [82]. However, the mechanisms remain understudied, and the mechanisms contributing to individual variance in menstrual fluctuations in anxiety are unknown and are difficult, time-intensive and costly to examine in humans. Studies in female rats offer a controlled laboratory setting to investigate the potential neurobiological and endocrinological mechanisms that may underlie menstrual-related changes in anxiety symptoms in humans with greater scale and pace. To help improve the translational value of female rat findings, in the following sections, we offer some considerations for future research.

### Defining and Aligning Oestrous and Menstrual Cycle Phases

Although there are broad similarities between the human and rat reproductive cycles (Fig. 1), there are also differences. In addition to differences in cycle length, the timing of ovulation relative to the progesterone surge differs between rats and humans, with ovulation occurring prior to the progesterone surge in humans and after the progesterone surge in rats. In both species, oestradiol surges prior to ovulation, but whereas humans experience a secondary rise in oestradiol during the post-ovulation luteal phase, this is absent in rats. Both species exhibit a post-ovulation rise in progesterone, but this appears to be stronger in humans than in rats. Finally, unlike humans, rats do not menstruate. As such, although the impact of relative differences in sex hormone levels on various behavioural outcomes can be compared in humans and rats, it is important to be mindful of cross-species differences when making inferences about humans based on rats – A point that is rarely acknowledged in studies on oestrous effects on anxiety-like behaviour.

We suggest that translational researchers justify their selection of the oestrous phase being examined and the time of day of testing based on the research question and indicate how it aligns with the human menstrual cycle. For example, if a researcher is interested in declining oestradiol and progesterone levels as experienced during the premenstrual phase in humans, this may correspond to the oestrus phase in rats. However, this may confound the impact of declining sex hormones with the period of sexual receptivity, so testing during metestrus or diestrus may be more appropriate. Some researchers use the afternoon of diestrus in female rats as an analogue to the premenstrual phase in humans, given that during diestrus, there is a withdrawal from a mini peak in progesterone which is then proceeded by a surge in oestradiol [73, 83–85]**.** Moreover, while some studies examine distinct oestrous phases [52–62, 68, 70–73], others combine phases with similar hormonal profiles, with proestrus and oestrus as a ‘high sex hormones’ group [51, 63–65, 66•, 67•, 69•, 74, 75] despite oestradiol and progesterone declining during oestrus and potentially diluting the anxiolytic effect of proestrus. Studying oestrous phases that are clearly defined with respect to hormonal state and are aligned to the human cycle will improve the translatability of rat findings to humans.

### Using Repeated Measures to Examine Individual Differences in Anxiety Across the Oestrous Cycle

**Fig. 2:**
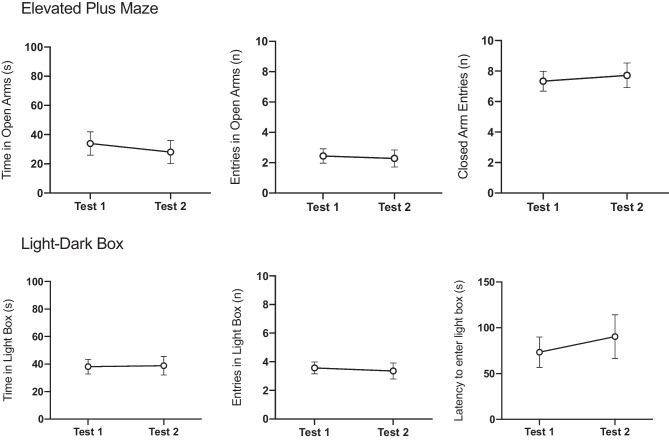
Unpublished findings from our lab**.** Sprague–Dawley adult female rats were tested twice on the elevated plus maze (*n* = 18) and twice on the light–dark box (*n* = 14). Half the rats were tested during metestrus first, while the other half were tested during proestrus first. There were no significant differences in any behaviour measures from test 1 to test 2 (paired-samples *t’*s < 1)

Whereas most rodent studies on oestrous effects adopt a between-subject design, most human studies on menstrual effects adopt a within-subject design, which, as noted, is better suited to detect menstrual-related changes in anxiety. Behaviour on unlearned fear tests in rats, such as the EPM, is commonly evaluated using a single trial to avoid behavioural changes that may arise due to non-associative learning processes (e.g. sensitization or habituation). This concern stems from the observation of a ‘one-trial tolerance’ to the anxiolytic-like effects of benzodiazepines on the EPM, wherein the anxiolytic effects are diminished upon a second exposure to the EPM [86, 87]. However, there is little evidence to suggest that sensitisation or habituation to anxiety tests occurs under non-drug-induced states. Rather, a recent study in male rats found that behaviour on the EPM was comparable over three trials, with deviations from the initial test only emerging in the fourth trial [88]. Unpublished data from our lab has found a similar lack of sensitisation or habituation in female rats over two testing trials on the EPM and LDB (Fig. 2). As such, repeated testing on unlearned fear tasks may be a valid approach for assessing oestrous effects on anxiety-like behaviour in female rats, requiring only two trials (e.g. once each during metestrus and proestrus), counterbalancing oestrous phase at the first test.

Examining oestrous effects on anxiety-like behaviour using a within-subjects design is advantageous because it allows researchers to identify underlying mechanisms that account for individual differences in oestrous-related exacerbation in anxiety-related behaviour, with the aim of translating these findings to understand the biological source of the heterogeneity of menstrual effects on anxiety in humans. Indeed, individual differences in oestrous effects on anxiety-like behaviour have been demonstrated on a marble burying task, such that only 30% out of 85 female rats exhibited cycle-dependent burying behaviour, with higher marble burying during metestrus versus proestrus [60]. Future studies could measure both anxiety-like behaviour (e.g. via EPM) and hormone/neurosteroid levels (e.g. blood collection via catheter) at multiple oestrous phases in the same rat. This experimental approach would enable researchers to identify whether *absolute* levels of hormones, *changes* in hormones and/or the *ratio* of hormones are associated with variability in oestrous effects on anxiety-like behaviour. Studies in rats could also identify the neurobiological processes underlying heightened sensitivity to hormonal fluctuations in the absence of differential hormone/neurosteroid levels. For example, unlike in humans, studies in rats could easily examine brain changes (e.g. neuronal activity via *cfos* expression, gene and protein expression) to typical hormonal changes across the oestrous cycle in rats identified as ‘hormone sensitive’ (i.e. rats that exhibit cyclic-dependent anxiety-like behaviour). This is important given recent demonstrations that cell cultures from people with versus without PMDD exhibit differential changes in gene expression following treatment with oestradiol and progesterone [89]. These findings highlight the need to examine cellular responsivity to hormones beyond the crude assessment of absolute changes in hormones, which is something that can be measured with greater ease in rats. The knowledge gained from such experiments may provide insights into the endocrinological and neurobiological characteristics of the subset of humans with and without anxiety disorders who experience menstrual fluctuations in anxiety.

### Modelling Multiple Features of Anxiety in Rats and Aligning Experimental Tasks Across Species

Just as anxiety symptoms in humans are comprised of affective, behavioural, physiological, and cognitive dimensions that can be assessed using different procedures, tests of unlearned fear in rats capture different facets of anxiety-like behaviour [90]. For example, some tasks measure active responses (burying behaviour, compulsive grooming), whereas others measure passive responses (freezing, avoidance). However, most studies measure anxiety-like behaviour on a single approach-avoidance task. While these tasks have high face validity given that avoidance is a key characteristic of anxiety disorders, the caveat of these tasks is that it is difficult to disentangle differences in emotionality and general locomotor activity, an issue which appears to be particularly relevant for female rats [91]. It has been suggested that researchers can address this post-hoc by calculating a ratio score of anxiety-like behaviours divided by locomotive measures (e.g. the ‘anxiety index’ [92, 93]). Another solution is to use a battery of tests, including active tasks, such as marble burying behaviour, acoustic startle response, light-potentiated startle response or social interaction, which would produce a more comprehensive picture of oestrous effects on anxiety [51, 56, 61, 62, 66•].

An advantage of using measures such as the acoustic startle response in female animals is that this test can also be examined in humans. Indeed, some studies report higher startle response during the late-luteal phase compared to the early follicular phase in humans [94, 95], although other studies report no changes [96, 97]. Another task that can be examined across species is the light-potentiated startle response in female rats and the dark-potentiated startle response in humans [98], yet no studies have examined the effect of the reproductive cycle on these tasks. Humans can also be tested on an EPM using virtual reality while also measuring physiological responses (e.g. heart rate, breathing) and subjective measures of anxiety [99, 100•]. Examining common tasks across species may improve the translatability of results. In addition, such findings will add to a growing body of research showing that cyclic sex hormones impact models of learned anxiety-related features in rats and humans, such as fear extinction (model of fear inhibition [101]) and pre-pulse inhibition of the startle response (model of sensorimotor gating [102]). Moreover, whereas rat studies assess anxiety-like behaviour under provoked (inherently threatening) situations, the majority of studies on menstrual-related effects on anxiety in healthy humans measure changes in basal (i.e. unprovoked) symptoms, with some notable exceptions [13, 46•]. More robust effects of the menstrual cycle could be unmasked in healthy people using tasks designed to provoke symptoms (e.g. behavioural approach tests with spiders, social stress paradigms); this is particularly relevant in healthy people, given that floor effects could contribute to null results.

### Testing Non-human Animals Selectively Bred for High Anxiety to Align with Clinically Anxious Humans

To the best of our knowledge, all research on oestrous effects on anxiety-like behaviour have been conducted using normally bred adult female rats, with one exception [65] that examined Wistar Kyoto rats, a strain associated with heightened stress sensitivity. Selective breeding for extremes in trait anxiety has produced rodent strains with inborn low or high anxiety-like behaviour [103, 104]. Whereas the literature on oestrous effects on anxiety in *normally bred* rats may offer insights into menstrual-related changes in anxiety in healthy people, future studies testing oestrous effects on anxiety-like behaviour in female rodents *selectively bred* for high anxiety-like behaviour may be a promising animal model for examining the genetic, neurobiological, and endocrinological causes of menstrual-related anxiety fluctuations in clinically anxious women. Additionally, examining oestrous effects on anxiety-like behaviour in both normally bred and high-anxiety rodents may be a useful analogue to examine potential differences in menstrual cycle effects on anxiety between healthy and clinically anxious human populations.

## Conclusions

Fluctuations in anxiety across the menstrual cycle are evident in both clinical and healthy human populations, although evidence to date indicates a more robust effect in clinical populations, and substantial heterogeneity is evident in both populations. Analogous fluctuations in anxiety-like behaviour across the oestrous cycle in rats point to an evolutionarily conserved biological mechanism that at least in part underlies these effects. Cross-species translational frameworks can be better leveraged to investigate and understand this effect. Future research should endeavour to improve the alignment of cross-species models through increasing the reliability and comparability of the hormonal phases that are examined, employing within-subject designs, and further investigating which individual factors may contribute to the incidence and pattern of menstrual-related fluctuations in anxiety across species.
